# Do Patients with Second Primary Colorectal Cancer Hold the Similar Prognosis and Therapeutic Benefits as Those with Initial Primary Colorectal Cancer?

**DOI:** 10.1155/2018/6172670

**Published:** 2018-08-30

**Authors:** Quan Chen, Shan Zhao, Yongxi Song, Peng Gao, Jingxu Sun, Xiaowan Chen, Yu Sun, Zhenning Wang

**Affiliations:** Department of Surgical Oncology and General Surgery, The First Hospital of China Medical University, 155 North Nanjing Street, Heping District, Shenyang City 110001, China

## Abstract

**Aim:**

The objective is to compare the differences on prognosis and the therapeutic benefits between initial and second primary colorectal cancer (pCRC).

**Methods:**

A dataset containing 377,271 initial pCRC cases and 18,617 second pCRC cases from the National Cancer Institute's Surveillance, Epidemiology, and End Results (SEER) 1988–2015 was evaluated. Survival comparisons were made using the log-rank test. Cox proportional hazards models were used to assess the survival benefits.

**Results:**

The cancer-specific survival rate of patients with initial pCRC was significantly higher than that of patients with second pCRC (5-years survival rate: 64.85% vs. 60.22%, P<0.001). The Chi-square of stratified log rank for age at diagnosis was lower than that for primary site, pTNM stage, sex, race, histology, and grade (Chi-square=86.73). There were almost no differences on therapeutic benefits between patients with initial and second pCRC except that treatments with chemotherapy were significantly associated with longer survival rate compared with treatments without chemotherapy among stage III surgical initial and second primary left-sided colon cancers patients (HR=0.764 vs. 0.581; P for interaction =0.008).

**Conclusion:**

Patients with second pCRC have worse prognosis than those with initial pCRC primarily because of older age in the former group. The results evidenced that the therapeutic benefits on the prognosis for colorectal cancer were generally similar between patients with initial and second pCRC.

## 1. Introduction

Colorectal cancer is the third most commonly diagnosed cancer and the leading cause of cancer-related mortality in the United States, with an incidence rate (2009–2013) of 40.7 per 100,000 persons and a mortality rate (2010–2014) of 14.8 per 100,000 persons [1, 2]. Early detection and therapies of patients with initial primary colorectal cancer (pCRC) have reduced the incidence and mortality rate, and the prognosis of patients with initial pCRC was improved by standard therapies involving surgery, radiotherapy (not in colon cancer patients), chemotherapy, neoadjuvant therapy, and adjuvant therapy [3, 4].

Recent studies have indicated that the incidence and risk of second primary cancers are increasing [5, 6], and this may possibly be due to the extended life expectancy of cancer patients [4]. Some studies [7, 8] reported that the majority of second primary cancers might occur at random and different mechanisms are involved, including the effects of therapies on the first cancer, environmental factors, and genetic predisposition. Colorectal cancer is one of the most prevalent second primary cancers [5]. Second pCRC is defined as second primary malignant colorectal cancers whose location or histology is different from that of initial pCRC and excludes the metastatic and recurrent lesions from the initial pCRC.

Second pCRC affects the same site but is anatomically distinct from the initial pCRC and thus is not a metastatic or recurrent tumor from the initial pCRC [7, 9–11]. With respect to the prognosis of patients with initial and second pCRC, Hildebrand et al. [11] observed that there were no significant differences on the prognosis of 1,500 patients with or without multiple primary colorectal cancers who underwent surgery, including those with second pCRC; however, the prognostic analysis was not based on a nationwide dataset or stratified by age at diagnosis and postoperative pathological stage (pTNM stage). Some clinical trials on therapies have been performed on patients with second pCRC [12]. Nevertheless, few studies to date evaluated the survival differences of the therapeutic effect between initial pCRC and second pCRC.

Therefore, the prognosis of second pCRC based on nationwide data compared with initial pCRC according to the pTNM stage, primary site, and age at diagnosis, and the therapeutic benefits on the prognosis of second pCRC is unclear. The objective of this study is to assess differences on prognosis and therapeutic effects between initial and second pCRC.

## 2. Materials and Methods

### 2.1. Data Source

The dataset from the National Cancer Institute's Surveillance, Epidemiology, and End Results (SEER) Program 1973–2015 contained incidence and population data. SEER data include cancer cases from various locations and sources across the United States and began to be collected in 1973 with a limited number of registries and the dataset continues to expand by including more geographical areas and demographics. The SEER dataset contains more than 10,050,814 entries, including 9,099,524 cases of malignancy and more than 700,000 cases of colorectal cancer. The patients diagnosed from 1988 to 2015 were included in the analysis. The primary study endpoint was cancer-specific survival (CSS), and comparisons of overall survival (OS) were presented in Supplementary Data. The registry is a tumor-based record, and second primary cancers in the same patients are registered separately. The SEER*∗*Stat software version 8.3.5 (IMS Inc. USA) was used to extract primary data.

### 2.2. Eligibility Criteria

The criteria for initial pCRC include only one primary site (International Classification of Diseases for Oncology, third edition (ICD-O-3) codes 18.0–18.7, 19.9, and 20.9) which was diagnosed.

The definition of second pCRC fulfills the following criteria: (1) interval between the diagnosis of initial primary cancers and second pCRC ≥5 years; (2) difference in the primary site between initial primary cancers and second pCRC; (3) histology being different if the primary site is the same as the primary site of the initial primary cancers. In accordance with clinical and surveillance follow-up criteria for second pCRC [8, 13–15], we considered defining the interval ≥5 years after diagnosis of initial primary cancers.

Patients were removed from this study in cases of (1) records of initial and second pCRC with missing data; (2) in situ tumors; (3) diagnosis before 1988 and death within 30 days after a confirmed diagnosis; (4) cases with histological ICD-O-3 codes, including 8000–8152, 8154–8231, 8243–8245, 8250–8576, 8940–8950, and 8980–8981[16]; (5) cases involving “intraoperative radiation,” “intraoperative radiation with other types of radiation before/after surgery,” “surgery both before and after radiation,” “sequence unknown, but both were given.”

### 2.3. Study Variables

Two groups of colorectal cancer patients were selected: one group with initial pCRC and another group with second pCRC whose second primary site was the colon or rectum. The evaluated cases were further categorized into three groups according to the primary site: right-sided colon (C18.0–18.4), left-sided colon (C18.5–18.7), and rectum (C19.9 or C20.9). The cases were staged by SEER's coding schemes “extent of disease” (for T and M categories) and “regional nodes positive” (for N category). Stage IV was regarded as an entirety because there was no evidence about the number of metastatic organs/sites in the SEER program [17]. The pTNM stage was classified according to the 8th edition of the Union for International Cancer Control or American Joint Committee on Cancer tumor-node-metastasis (TNM) staging system [16]. Patients subjected to “beam radiation,” “radioactive implants,” “radioisotopes,” “beam radiation combined with implants or isotopes,” and “radiation, NOS method or unspecified sources” were categorized as radiotherapy performed. The patients with unknown therapy delivery were assigned to the category “None/Unknown.”

### 2.4. Statistical Analysis

Categorical variables were compared using the *χ*^2^ test. The CSS and OS were determined using Kaplan–Meier survival curves and compared using the log-rank test. Prognosis and the therapeutic effect were analyzed using subgroup stratified analyses and presented by the Chi-square of stratified log rank [18]. Cox proportional hazards models were constructed to assess the therapies for initial and second pCRC using adjusted hazard ratio (HR) and 95% confidence interval (CI). The tests used for establishing correlations were conducted by adding an interaction term to the model, and likelihood ratio tests were used to determine the statistical significance from Cox proportional hazards model.

Statistical analyses were performed using software IBM SPSS Statistics version 20 (IBM Corporation, Armonk, NY, USA) and STATA MP version 14 (StataCorp LP, College Station, TX, USA). A P value < 0.050 was considered statistically significant.

## 3. Results

### 3.1. Characteristics of Initial and Second pCRC

We obtained one dataset consisting of 377,271 cases of initial pCRC and 18,617 cases of second pCRC. The characteristics of pCRC and the *χ*^2^ test for comparison of initial and second pCRC are shown in Table 1. There were significant differences in gender, race, primary site, histology, grade, pT, pN, pM, surgery, radiation, and chemotherapy (P<0.001 each) between patients with initial and second pCRC. Compared with patients with initial pCRC, patients with second pCRC were more often diagnosed with age≥70 years (73.73% vs. 45.08%), pTNM stage I and II (55.25% vs. 47.28%), and right-sided colon cancer (53.48% vs. 44.23%).

### 3.2. CSS Comparison between Patients with Initial and Second pCRC

**Figure 1:**
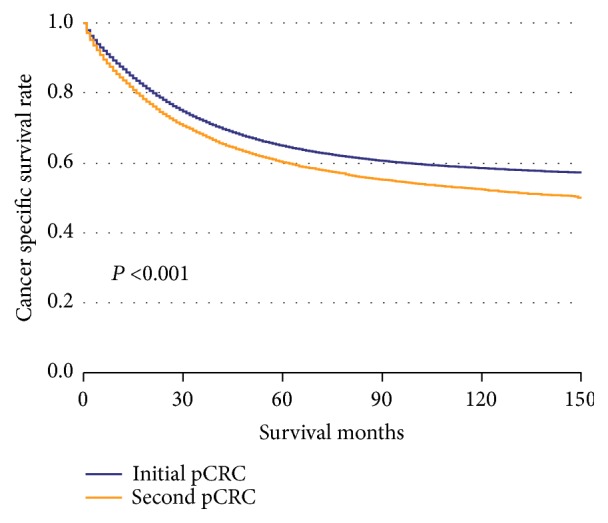
Kaplan–Meier comparison of cancer-specific survival among patients with initial pCRC and second pCRC.

**Figure 2:**
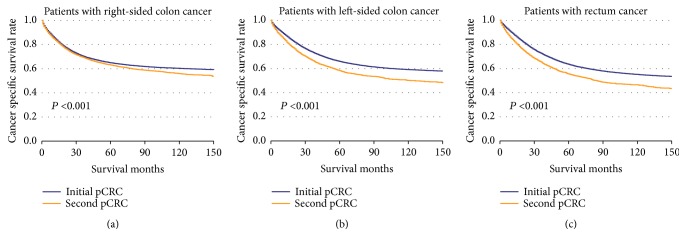
**Kaplan–Meier comparison of cancer-specific survival among patients with initial pCRC and second pCRC stratified by primary site.** (a) Patients with right-sided colon cancer (initial pCRC vs. second pCRC); (b) patients with left-sided colon cancer (initial pCRC vs. second pCRC); (c) patients with rectum cancer (initial pCRC vs. second pCRC).

**Figure 3:**
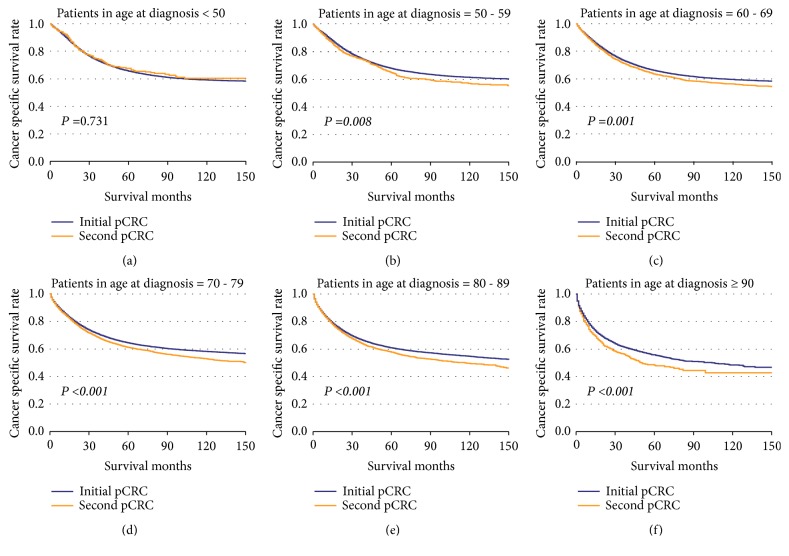
**Kaplan–Meier comparison of cancer-specific survival among patients with initial pCRC and second pCRC stratified by age at diagnosis.** (a) Patients in age at diagnosis > 50 (initial pCRC vs. second pCRC); (b) patients in age at diagnosis = 50 - 59 (initial pCRC vs. second pCRC); (c) patients in age at diagnosis = 60 - 69 (initial pCRC vs. second pCRC); (d) patients in age at diagnosis = 70 - 79 (initial pCRC vs. second pCRC); (e) patients in age at diagnosis = 80 - 89 (initial pCRC vs. second pCRC); (f) patients in age at diagnosis ≥ 90 (initial pCRC vs. second pCRC).

**Figure 4:**
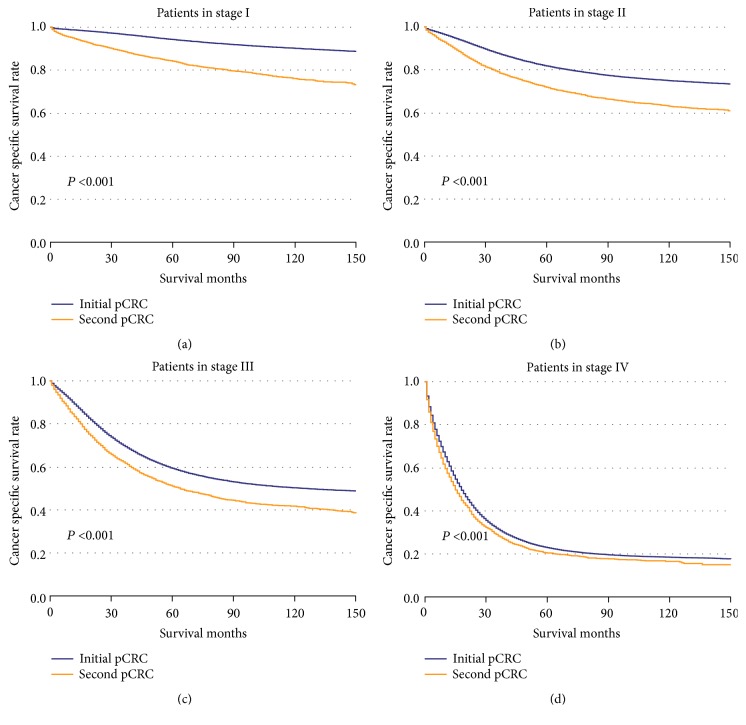
**Kaplan–Meier comparison of cancer-specific survival among patients with initial pCRC and second pCRC stratified by pTNM stage.** (a) Patients in stage I (initial pCRC vs. second pCRC); (b) patients in stage II (initial pCRC vs. second pCRC); (c) patients in stage III (initial pCRC vs. second pCRC); (d) patients in stage IV (initial pCRC vs. second pCRC).

**Figure 5:**
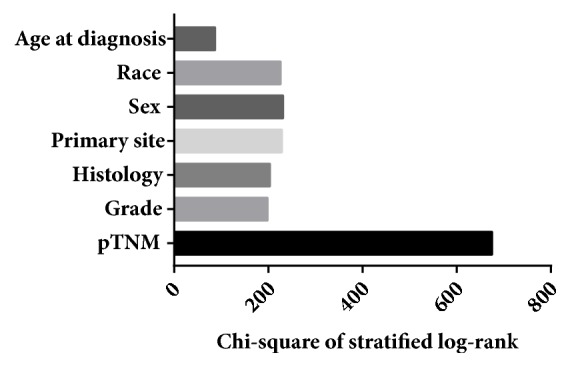
Chi-square of CSS log rank after stratified by pTNM stage, grade, histology, primary site, sex, race, and age at diagnosis.

The CSS of patients with initial pCRC was significantly higher than that of patients with second pCRC (5-year survival rate [5-YSR]: 64.85% versus 60.22%, P<0.001; Figure 1). Patients were stratified according to primary site (Figure 2), age at diagnosis (Figure 3), and pTNM stage (Figure 4). In the subgroups with significant differences, the 5-YSR of patients with initial pCRC was consistently and significantly higher than that of patients with second pCRC. This association persisted when the groups were stratified by primary site (Figure 2) and pTNM stage (Figure 4). However, there was no significant difference in age at diagnosis <50 years between the initial and second pCRC (P=0.731; Figure 3(a)). In the stratified log-rank test, the Chi-square for age at diagnosis was lower (Chi-square=86.73; Figure 5) than that for primary site, pTNM stage, sex, histology, and grade. The same comparisons were performed using OS (Supplemental Data–eFigures 1–4), and the Chi-square of OS log rank after stratification by age at diagnosis was also lower than the others (Chi-square=131.43; Supplemental Data–eFigure 5).

A similar result was obtained after modeling the above variables in multivariate analysis (HR=1.162; 95% CI=1.133-1.192; P<0.001; Table 2). Meanwhile, when analyzed by OS, patients with second pCRC hold worse OS than those with initial pCRC (HR=1.102; 95% CI=1.081-1.123; P<0.001; Supplemental Data–eTable 1).

### 3.3. Benefits of Different Therapies for Initial and Second pCRC

**Figure 6:**
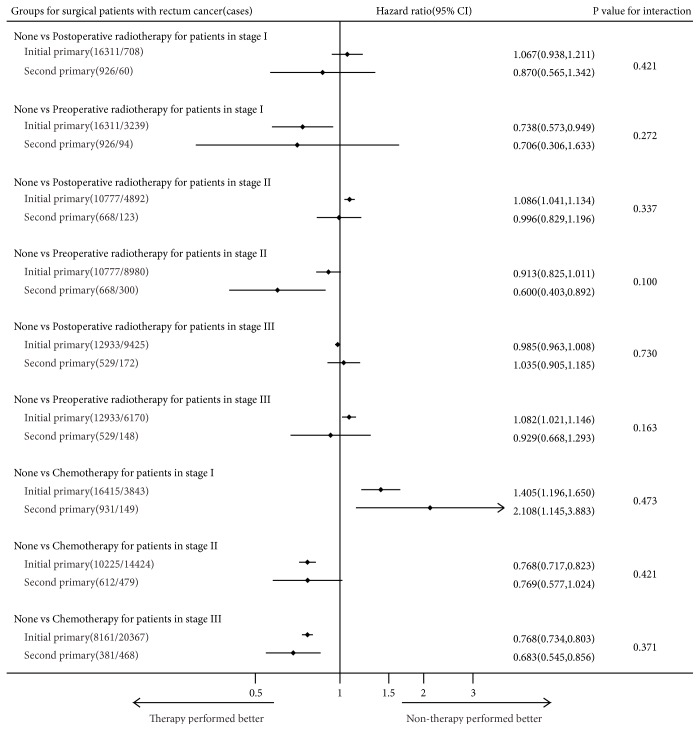
Forest plot of hazard ratios (HR) and 95% CI in CSS different subgroups (Cox proportional hazards model analysis) and interaction comparison between patients with initial and second primary surgical rectum cancer.

**Figure 7:**
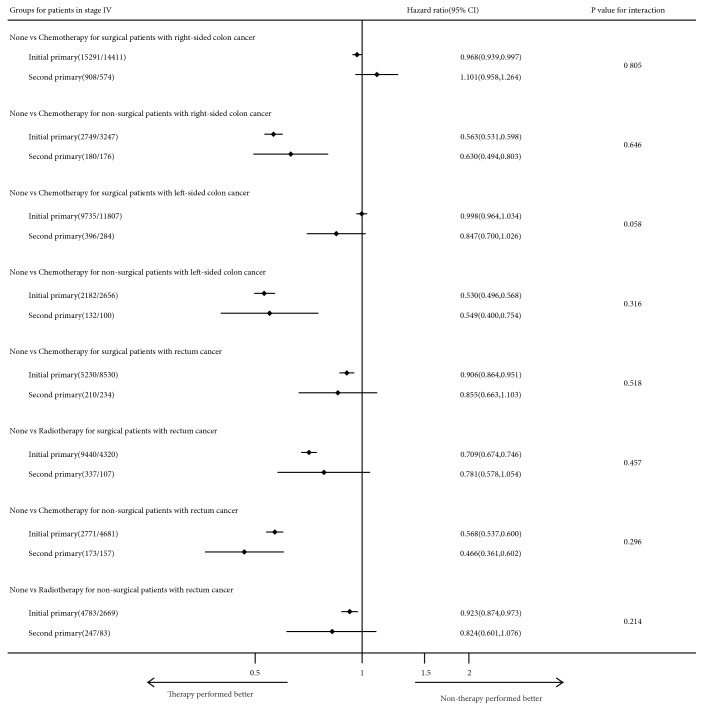
Forest plot of hazard ratios (HR) and 95% CI in CSS different subgroups (Cox proportional hazards model analysis) and interaction comparison between patients in stage IV with initial and second primary CRC.

**Figure 8:**
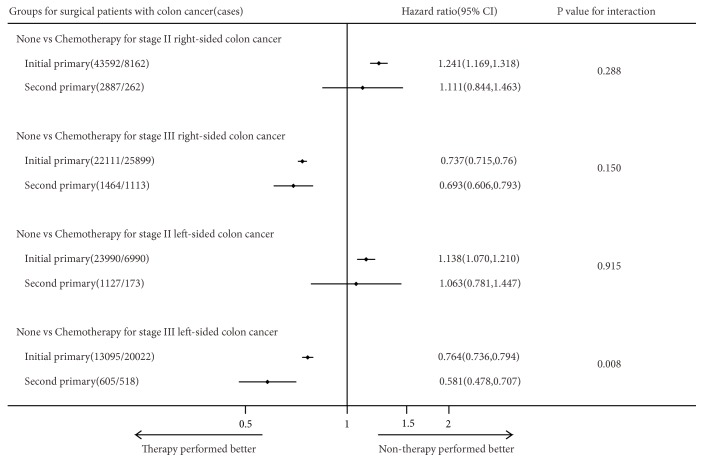
Forest plot of hazard ratios (HR) and 95% CI in CSS different subgroups (Cox proportional hazards model analysis) and interaction comparison between patients with initial and second primary surgical colon cancer.

We excluded the patients in stages I, II, and III who were not subjected to surgery but kept the patients in stage IV without surgery. There were 65,004 (77.05%) initial pCRC and 2,606 (73.95%) second pCRC stage IV surgical patients and 18,286 (22.95%) initial pCRC and 918 (26.05%) second pCRC stage IV none/unknown surgical patients (*χ*^2^-test P <0.001). The benefits of therapies for initial and second pCRC were evaluated by performing a multivariate analysis on CSS stratified by primary site and pTNM stage and using interactions to assess the difference between primary sequence and the benefits of therapies (Figures 6–8).

Among surgical patients in stage III with initial primary left-sided colon cancers, treatments with chemotherapy were significantly associated with longer CSS compared with treatments without chemotherapy (HR=0.764; 95% CI=0.736–0.794; P<0.001). Similarly, among surgical patients in stage III with second primary left-sided colon cancers, treatments with chemotherapy were strongly associated with longer CSS compared with treatments without chemotherapy (HR=0.581; 95% CI=0.478–0.707; P<0.001). There was a significant interaction effect between primary sequence and the efficacy of chemotherapy among surgical patients in stage III with second primary left-sided colon cancers (P for interaction=0.008; Figure 8). However, except for this, there were no significant interactions effects found in the tests of interactions (Figures 6–8). The same analysis was performed using OS (Supplemental Data–eFigures 6–8), and treatments with chemotherapy were significantly associated with longer survival compared with treatments without chemotherapy among stage III surgical initial and second primary left-sided colon cancers patients (HR= 0.703 vs. 0.545) and there was also a significant interaction effect (P for interaction=0.026; Supplemental Data–eFigure 8).

## 4. Discussion

Second pCRC is not rare in the clinical setting, and the 5-year cumulative incidence of second pCRC is 2.1% [19, 20]. However, few studies to date evaluated the differences on prognosis and therapeutic benefits between initial and second pCRC using a nationwide database.

With respect to the pTNM stage, 24.46% and 30.79% of patients with second pCRC were in stages I and II, respectively, versus 18.77% and 28.51% of patients with initial pCRC, respectively. This result differs from that of previous studies, whereby most patients with second pCRC were in advanced stages [21, 22]. This difference may be because patients with second pCRC have early follow-ups and advanced examinations after diagnosis of cancer.

Our results indicated that 36.35% and 31.68% of patients with second pCRC were diagnosed at the ages of 70–79 and 80–89 years, respectively, whereas 24.42% and 25.83% of patients with initial pCRC were diagnosed at the ages of 60–69 and 70–79 years, respectively, demonstrating that patients with second pCRC were in older age groups at diagnosis compared to patients with initial pCRC, and the result agrees with previous studies [22, 23]. This difference may be due to the extended life expectancy of cancer patients after the effective therapies of the initial cancers. However, some studies found that there was no significant difference in age at diagnosis between patients with initial and second pCRC [21, 24, 25].

Hildebrand et al. [11] analyzed the survival prognosis between patients with initial and second pCRC after stratification by age at diagnosis (<60 years and >60 years) and found that there were no significant differences in this variable among patients with second pCRC. Some studies [26, 27] focused on patients with initial pCRC and multiple primary colorectal cancers and found that prognosis was similar between these two groups. In contrast, we found that patients with second pCRC had worse prognosis compared with patients with initial pCRC. This disagreement between studies may contribute to the differences of databases used. Considering SEER program was a nationwide database, we could obtain more patients with second pCRC and make stratification into more detailed subgroups for analysis. Rennert et al. [21] reported that the TNM stage of patients with second pCRC was a leading cause of adverse prognosis because most of these patients were diagnosed in advanced stages. In contrast, most patients with second pCRC in our study were in stages I and II and had worse survival rates.

Stratification by primary site, pTNM stage, age at diagnosis, sex, histology, and grade using the log-rank test indicated that age at diagnosis was the main independent factor leading to the differences in survival (Figure 5, Supplemental Data – eFigure 5). Moreover, the stratification indicated that patients with second pCRC were diagnosed at an older age compared to patients with initial pCRC and the former group had worse prognosis.

Surgery was considered the first choice in patients with initial and second pCRC for improving the survival rate [4, 22, 24]. Therefore, the patients in stages I, II, and III not subjected to surgery were excluded from this analysis when evaluated benefits of chemotherapy and radiotherapy on survival, while the patients in stage IV not subjected to surgery were included. With regard to the patients with colon cancers, the survival rate did not change significantly after they were subjected to radiotherapy [4]; therefore we only compared the survival differences in patients with colon cancer subjected to chemotherapy.

Kumar et al. [28] reported that there were significant differences in survival in patients with stages II and III multiprimary colorectal cancer undergoing adjuvant therapy and contributed to patients subjected to adjuvant therapy with older age and comorbidities. However, in our study, age at diagnosis was included in the multivariate analysis in the Cox regression model, and the interval between diagnoses of initial and second pCRC <5 years was excluded to ensure that the secondary lesions were not the comorbidities of the initial primary cancers.

The results evidenced that the therapeutic benefits on the prognosis for colorectal cancer were generally similar between the initial and the second pCRC (Figures 6–8). While for surgical patients suffered stage III right-sided colon cancer, treatments with chemotherapy were significantly associated with longer CSS compared with treatments without chemotherapy, there was no difference between two groups (P for interaction=0.150). However, we found significant difference between the two groups considering patients with stage III left-sided colon cancer (P for interaction=0.008). In this respect, some studies [29, 30] showed that patients with left-sided colon cancers had longer survival after using target and chemotherapy drugs and presented a higher sensitivity to bevacizumab treatment compared with patients with right-sided colon cancers. These apparent differences between left-sided and right-sided colon cancers may be because, at the molecular level, left-sided colon cancers have better predictive markers for patients undergoing chemotherapy, such as CIN, p53, and NRAS mutations.

This study has limitations. First, the study design was retrospective. Although SEER provides a platform for in-depth longitudinal analysis of cancer patients, it is still considered administrative data and thus subject to the standard bias and lack of granularity associated with large administrative databases. Second, due to the limited SEER dataset, disease-free survival status of patients with pCRC cannot be included in this study. Third, clinical datums related to prognosis, including karnofsky performance score (PS score), dosage and frequency of radiation, target therapy record, metastasis site, and the interval and sequence between chemotherapy and surgery, could not be investigated because these characteristics were not available in the SEER database. Moreover, the initial pCRC patients data we used for analysis in this study may potentially develop a second pCRC after the initial primary because the survival follow-up of some patients diagnosed recently was not long. This might underestimate the number of cases of second pCRC.

## 5. Conclusion

Patients with second pCRC have worse prognosis than those with initial pCRC primarily because of older age in the former group. The results evidenced that the therapeutic benefits on the prognosis for colorectal cancer were generally similar between patients with initial and second pCRC.

## Figures and Tables

**Table 1:** Characteristics of initial and second pCRC.

	Case number (%)		P^*∗*^
	Initial pCRC	Second pCRC	
Gender			<0.001
Male	188815(50.05%)	9806(52.67%)	
Female	188456(49.95%)	8811(47.33%)	
Age at diagnosis			<0.001
<50	43365(11.49%)	430(2.31%)	
50-59	71720(19.01%)	1278(6.86%)	
60-69	92114(24.42%)	3183(17.10%)	
70-79	97457(25.83%)	6768(36.35%)	
80-89	63222(16.76%)	5897(31.68%)	
≥90	9393(2.49%)	1061(5.70%)	
Race			<0.001
White	302958(80.30%)	15782(84.77%)	
Black	40759(10.80%)	1721(9.24%)	
Other†	32573(8.63%)	1105(5.94%)	
Unknown	981(0.27%)	9(0.05%)	
Primary site			<0.001
Right colon	166879(44.23%)	9957(53.48%)	
Left colon	114234(30.28%)	4522(24.29%)	
Rectum	96158(25.49%)	4138(22.23%)	
Histology			<0.001
AC	333711(88.45%)	16146(86.73%)	
MC	36328(9.63%)	1969(10.58%)	
SRCC	3890(1.03%)	222(1.19%)	
Other	3342(0.89%)	280(1.50%)	
Grade‡			<0.001
Grade I	29746(7.88%)	1483(7.97%)	
Grade II	249448(66.12%)	11913 (63.99%)	
Grade III	67017(17.77%)	3363(18.06%)	
Grade IV	5936(1.57%)	365(1.96%)	
Unknown	25124(6.66%)	1493(8.02%)	
pT			<0.001
Tis	6129(1.62%)	264(1.42%)	
T1	41027(10.87%)	2716(14.59%)	
T2	50839(13.48%)	2721(14.62%)	
T3	188967(50.09%)	8805(47.29%)	
T4a	26450(7.01%)	1261(6.77%)	
T4b	21062(5.58%)	959(5.15%)	
Unknown	42797(11.35%)	1891(10.16%)	
pN			<0.001
N0	202070(53.56%)	11359(61.01%)	
N1a	41901(11.11%)	1735(9.32%)	
N1b	45591(12.09%)	1806(9.70%)	
N2a	32414(8.59%)	1284(6.90%)	
N2b	30613(8.11%)	1134(6.09%)	
Unknown	24682(6.54%)	1299(6.98%)	
pM			<0.001
M0	293981(77.92%)	15093(81.07%)	
M1	83290(22.08%)	3524(18.93%)	
pTNM stage§			<0.001
0	5732(1.52%)	255(1.37%)	
I	70797(18.77%)	4553(24.46%)	
IIA	91890(24.35%)	4898(26.31%)	
IIB	8667(2.30%)	463(2.49%)	
IIC	7025(1.86%)	371(1.99%)	
IIIA	12095(3.20%)	472(2.54%)	
IIIB	71106(18.85%)	2959(15.89%)	
IIIC	26669(7.07%)	1122(6.03%)	
IV	83290(22.08%)	3524(18.92%)	
Surgery			<0.001
Performed	358475(95.02%)	17165(92.20%)	
None/Unknown	18796(4.98%)	1452(7.80%)	
Radiotherapy sequence			<0.001
Before surgery	21472(5.69%)	614(3.30%)	
After surgery	22464(5.96%)	552(2.96%)	
None/Unknown	333335(88.35%)	17451(93.74%)	
Radiotherapy			<0.001
Performed	47092(12.48%)	1424(7.65%)	
None/Unknown	330179(87.52%)	17193(92.35%)	
Chemotherapy			<0.001
Performed	146029(38.71%)	4907(26.36%)	
None/Unknown	231242(61.29%)	13710(73.64%)	

*Abbreviations.* AC adenocarcinoma, MC Mucinous adenocarcinoma, and SRCC Signet ring cell carcinoma.

*∗* P values were made by *χ*^2^-test.

† Other=American Indian/AK Native, and Asian/Pacific Islander, according to SEER.

‡ Grade I = well differentiated; grade II = moderately differentiated; grade III = poorly differentiated; grade IV = undifferentiated; anaplastic.

§ pTNM stage according to the 8th edition of the Union for International Cancer Control or American Joint Committee on Cancer tumor-node-metastasis (TNM) staging system.

**Table 2:** Prognostic factors in cox proportional hazard model (CSS).

	Univariate Analysis			Multivariate Analysis		
	HR	95%CI	P	HR	95%CI	P
Group			<0.001			<0.001
Initial pCRC	1			1		
Second pCRC	1.210	1.180-1.240		1.162	1.133-1.192	
Gender			<0.001			<0.001
Male	1			1		
Female	0.933	0.923-0.943		0.904	0.894-0.913	
Age at diagnosis			<0.001			<0.001
<50	1			1		
50-59	0.933	0.914-0.953		1.114	1.092-1.138	
60-69	1.020	1.000-1.040		1.315	1.290-1.341	
70-79	1.111	1.090-1.132		1.606	1.575-1.638	
80-89	1.294	1.268-1.320		2.045	2.001-2.090	
≥90	1.652	1.595-1.712		2.630	2.535-2.729	
Race			<0.001			<0.001
White	1			1		
Black	1.243	1.223-1.264		1.252	1.232-1.273	
Other†	0.886	0.869-0.904		0.884	0.867-0.902	
Unknown	0.266	0.216-0.327		0.368	0.299-0.453	
Primary Site			<0.001			<0.001
Right colon	1			1		
Left colon	0.957	0.945-0.970		1.006	0.993-1.019	
Rectum	1.050	1.036-1.064		1.061	1.045-1.078	
Histology			<0.001			<0.001
AC	1			1		
MC	1.245	1.224-1.266		1.022	1.005-1.040	
SRCC	2.716	2.611-2.825		1.245	1.196-1.296	
Other	2.446	2.338-2.559		1.090	1.041-1.141	
Grade‡			<0.001			<0.001
Grade I	1			1		
Grade II	1.525	1.488-1.563		1.116	1.088-1.144	
Grade III	2.852	2.778-2.927		1.423	1.385-1.461	
Grade IV	3.050	2.917-3.190		1.538	1.470-1.610	
Unknown	2.829	2.746-2.915		1.211	1.174-1.250	
pT			<0.001			<0.001
Tis	0.138	0.124-0.154		0.220	0.197-0.246	
T1	0.283	0.274-0.292		0.414	0.401-0.427	
T2	0.370	0.361-0.379		0.500	0.488-0.513	
T3	1			1		
T4a	1.906	1.870-1.943		1.577	1.546-1.608	
T4b	2.932	2.877-2.989		2.286	2.241-2.331	
Unknown	7.818	7.715-7.922		2.513	2.465-2.561	
pN			<0.001			<0.001
N0	1			1		
N1a	2.344	2.300-2.389		1.938	1.901-1.977	
N1b	3.350	3.293-3.407		2.512	2.467-2.557	
N2a	4.850	4.766-4.937		3.272	3.211-3.335	
N2b	7.376	7.252-7.503		4.438	4.354-4.523	
Unknown	17.801	17.493-18.115		2.985	2.895-3.078	
pM			<0.001			<0.001
M0	1			1		
M1	5.636	5.575-5.698		2.216	2.181-2.252	
Surgery			<0.001			<0.001
None/Unknown	1			1		
Performed	0.122	0.120-0.124		0.541	0.525-0.558	
Radiotherapy sequence			<0.001			<0.001
None/Unknown	1			1		
Before surgery	0.745	0.726-0.765		1.164	1.130-1.198	
After surgery	1.178	1.155-1.202		1.157	1.131-1.183	
Chemotherapy			<0.001			<0.001
None/Unknown	1			1		
Performed	1.677	1.660-1.695		0.799	0.789-0.809	

*Abbreviations.* AC Adenocarcinoma, MC Mucinous adenocarcinoma, SRCC Signet ring cell carcinoma.

† other=American Indian/AK Native, Asian/Pacific Islander, according to SEER.

‡ grade I = well differentiated; grade II = moderately differentiated; grade III = poorly differentiated; grade IV = undifferentiated; anaplastic.

## Data Availability

The dataset generated and analyzed during the current study is available in the National Cancer Institute's Surveillance, Epidemiology, and End Results SEER repository [https://seer.cancer.gov/].

## Abbreviations

pCRC: Primary colorectal cancer
SEER: Surveillance, Epidemiology, and End Results
TNM: Tumor-node-metastasis
pTNM stage: Postoperative pathological stage
AJCC: American Joint Committee on Cancer
CSS: Cancer-specific survival
OS: Overall survival
5-YSR: 5-year survival rate
HR: Hazard ratio
CI: Confidence interval
AC: Adenocarcinoma
MC: Mucinous adenocarcinoma
SRCC: Signet ring cell carcinoma
PS score: Karnofsky performance score.

## Supplementary Materials

Supplementary Materials
** eTable** **1.** Prognostic factors in Cox proportional hazard model (overall survival).** eFigure ****1** Kaplan-Meier comparison of overall survival among patients with initial pCRC and second pCRC.** eFigure ****2** Kaplan–Meier comparison of overall survival among patients with initial pCRC and second pCRC stratified by primary site.** eFigure ****3** Kaplan–Meier comparison of overall survival among patients with initial pCRC and second pCRC stratified by age at diagnosis.** eFigure ****4** Kaplan–Meier comparison of overall survival among patients with initial pCRC and second pCRC stratified by pTNM stage.** eFigure ****5** Chi-square of OS log rank after stratified by pTNM stage, grade, histology, primary site, sex, race, and age at diagnosis.** eFigure ****6** Forest plot of hazard ratios (HR) and 95% CI in OS different subgroups (Cox proportional hazards model analysis) and interaction comparison between patients with initial and second primary surgical rectum cancer.** eFigure ****7** Forest plot of hazard ratios (HR) and 95% CI in OS different subgroups (Cox proportional hazards model analysis) and interaction comparison between patients in stage IV with initial and second primary CRC.** eFigure ****8** Forest plot of hazard ratios (HR) and 95% CI in OS different subgroups (Cox proportional hazards model analysis) and interaction comparison between patients with initial and second primary surgical colon cancer.
