# Supplementary material for: Do Patients with Second Primary Colorectal Cancer Hold the Similar Prognosis and Therapeutic Benefits as Those with Initial Primary Colorectal Cancer?
Source: Biomed Res Int. 2018 Aug 30;2018:6172670. doi: 10.1155/2018/6172670 (PMC6136497; doi:10.1155/2018/6172670)
Supplement: Supplementary Materials — eTable 1. Prognostic factors in Cox proportional hazard model (overall survival). eFigure 1 Kaplan-Meier comparison of overall survival among patients with initial pCRC and second pCRC. eFigure 2 Kaplan–Meier comparison of overall survival among patients with initial pCRC and second pCRC stratified by primary site. eFigure 3 Kaplan–Meier comparison of overall survival among patients with initial pCRC and second pCRC stratified by age at diagnosis. eFigure 4 Kaplan–Meier comparison of overall survival among patients with initial pCRC and second pCRC stratified by pTNM stage. eFigure 5 Chi-square of OS log rank after stratified by pTNM stage, grade, histology, primary site, sex, race, and age at diagnosis. eFigure 6 Forest plot of hazard ratios (HR) and 95% CI in OS different subgroups (Cox proportional hazards model analysis) and interaction comparison between patients with initial and second primary surgical rectum cancer. eFigure 7 Forest plot of hazard ratios (HR) and 95% CI in OS different subgroups (Cox proportional hazards model analysis) and interaction comparison between patients in stage IV with initial and second primary CRC. eFigure 8 Forest plot of hazard ratios (HR) and 95% CI in OS different subgroups (Cox proportional hazards model analysis) and interaction comparison between patients with initial and second primary surgical colon cancer. [file 6172670.f1.pdf]

Supplemental Data

eTable1. Prognostic factors in cox proportional hazard model(Overall survival)

	Univariate Analysis			Multivariate Analysis		
	HR	95%CI	p	HR	95%CI	P
Group			<0.001			<0.001
Initial pCRC	1			1		
Second pCRC	1.384	1.358-1.410		1.102	1.081-1.123	
Gender			<0.001			<0.001
Male	1			1		
Female	0.962	0.954-0.970		0.845	0.838-0.852	
Age at diagnosis			<0.001			<0.001
<50	1			1		
50-59	1.034	1.014-1.054		1.158	1.136-1.180	
60-69	1.352	1.328-1.376		1.590	1.561-1.618	
70-79	1.972	1.939-2.006		2.468	2.426-2.512	
80-89	2.984	2.932-3.036		3.913	3.841-3.986	
≥90	4.393	4.281-4.508		5.770	5.617-5.928	
Race			<0.001			<0.001
White	1			1		
Black	1.100	1.085-1.115		1.224	1.207-1.241	
Other†	0.802	0.789-0.815		0.847	0.834-0.861	
Unknown	0.257	0.218-0.302		0.345	0.293-0.406	
Primary Site			<0.001			<0.001
Right colon	1			1		
Left colon	0.856	0.848-0.865		0.997	0.987-1.007	
Rectum	0.862	0.853-0.871		1.041	1.029-1.054	
Histology			<0.001			<0.001
AC	1			1		
MC	1.206	1.191-1.223		1.017	1.004-1.031	
SRCC	2.197	2.120-2.276		1.313	1.266-1.361	
Other	2.004	1.924-2.087		1.051	1.009-1.096	
Grade‡			<0.001			<0.001
Grade I	1			1		
Grade II	1.230	1.209-1.251		1.040	1.022-1.057	
Grade III	1.898	1.864-1.934		1.225	1.202-1.249	
Grade IV	2.070	1.996-2.148		1.340	1.291-1.391	
Unknown	1.807	1.767-1.847		1.093	1.068-1.119	
pT			<0.001			<0.001
Tis	0.469	0.449-0.489		0.589	0.563-0.615	
T1	0.521	0.512-0.530		0.650	0.638-0.662	
T2	0.656	0.647-0.666		0.748	0.737-0.759	
T3	1			1		

T4a	1.539	1.515-1.563		1.383	1.361-1.405	
T4b	2.127	2.092-2.163		1.934	1.901-1.967	
Unknown	5.368	5.306-5.431		2.293	2.253-2.334	
pN			<0.001			<0.001
N0	1			1		
N1a	1.411	1.391-1.431		1.384	1.364-1.405	
N1b	1.782	1.759-1.806		1.677	1.654-1.700	
N2a	2.384	2.350-2.418		2.152	2.119-2.185	
N2b	3.498	3.449-3.548		3.006	2.959-3.054	
Unknown	8.621	8.493-8.750		2.118	2.060-2.177	
pM			<0.001			<0.001
M0	1			1		
M1	3.593	3.560-3.627		1.868	1.842-1.894	
Surgery			<0.001			<0.001
None/Unknown	1			1		
Performed	0.159	0.157-0.162		0.537	0.522-0.552	
Radiotherapy sequence			<0.001			<0.001
None/Unknown	1			1		
Before surgery	0.625	0.611-0.639		1.063	1.037-1.090	
After surgery	0.903	0.888-0.919		1.061	1.041-1.082	
Chemotherapy			<0.001			<0.001
None/Unknown	1			1		
Performed	1.045	1.036-1.054		0.769	0.761-0.777	

Abbreviation: AC Adenocarcinoma, MC Mucinous adenocarcinoma, SRCC Signet ring cell carcinoma

† other=American Indian/AK Native, Asian/Pacific Islander, according to SEER

‡ grade I = well differentiated; grade II = moderately differentiated; grade III = poorly differentiated; grade IV = undifferentiated; anaplastic

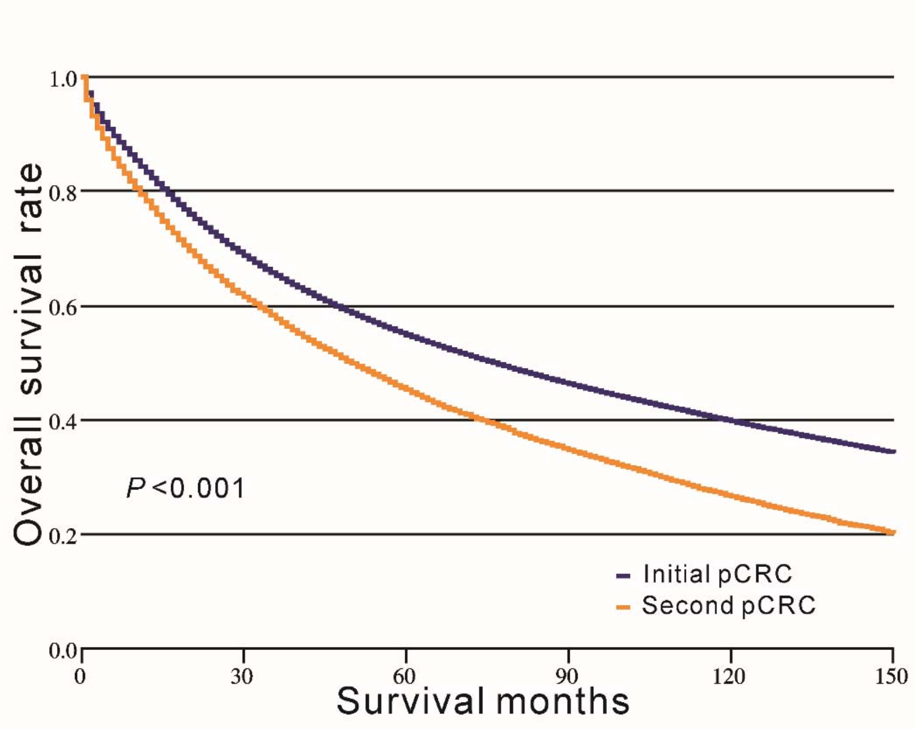

eFigure 1 Kaplan-Meier comparison of overall survival among patients with initial pCRC and second pCRC.

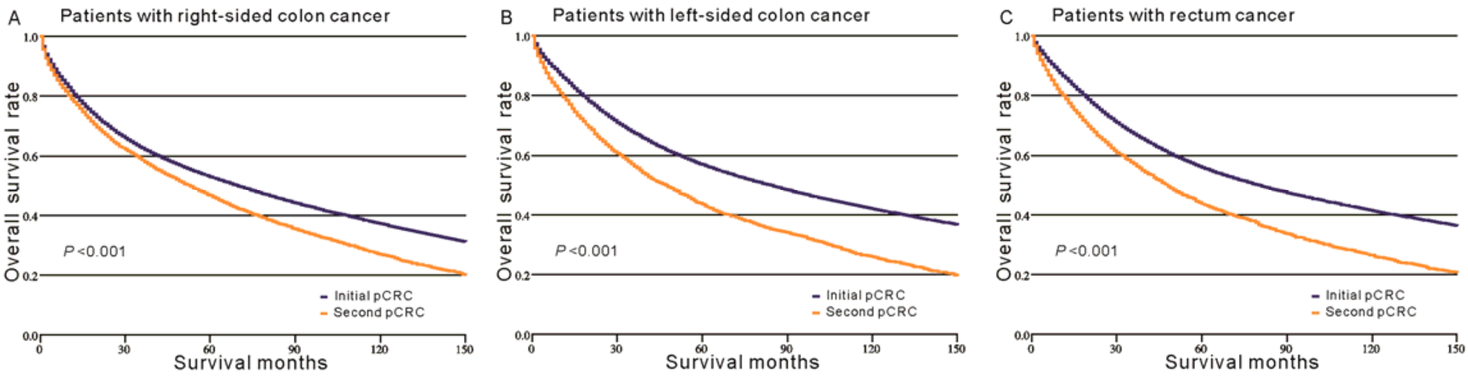

Figure 2 Kaplan-Meier comparison of overall survival among patients with initial pCRC and second pCRC stratified by primary site.

(A) Patients with right-sided colon cancer (initial pCRC vs. second pCRC); (B) Patients with left-sided colon cancer (initial pCRC vs. second pCRC); (C) Patients with rectum cancer (initial pCRC vs. second pCRC).

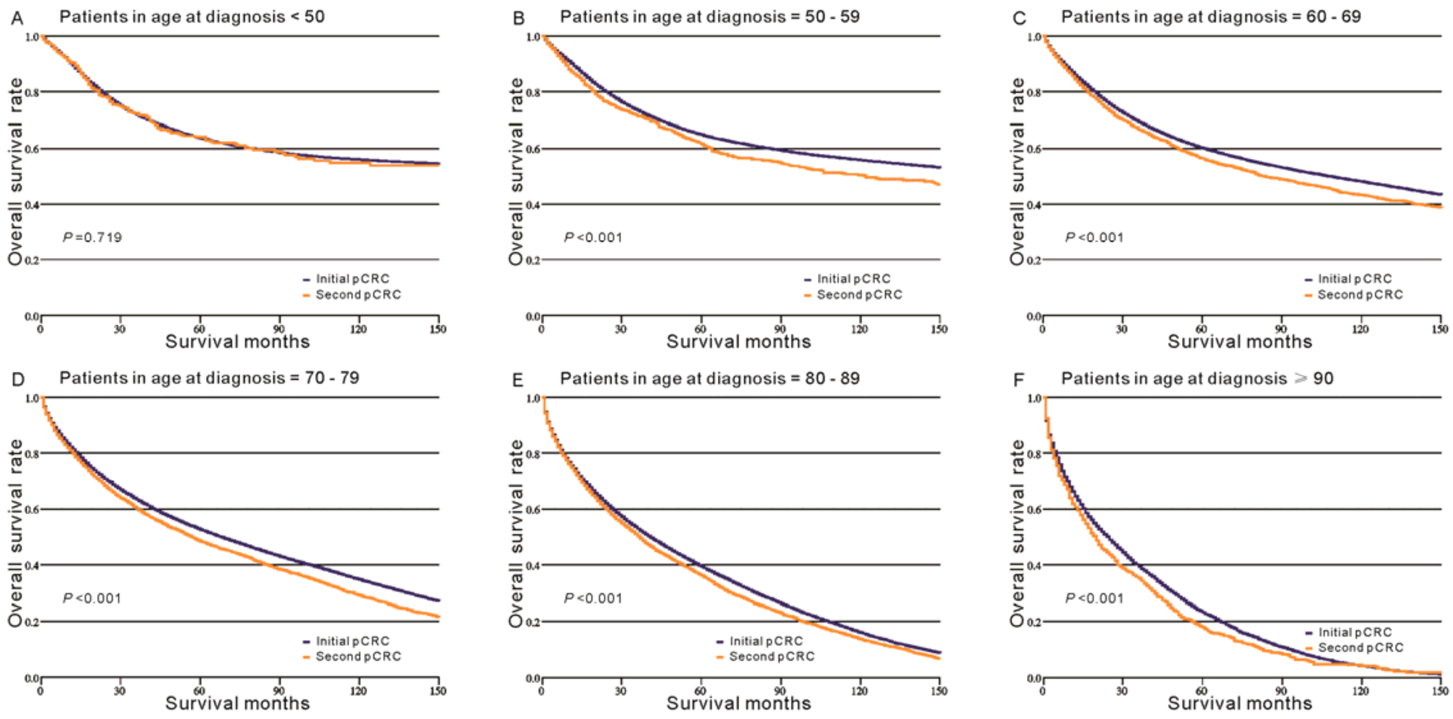

Figure 3 Kaplan-Meier comparison of overall survival among patients with initial pCRC and second pCRC stratified by age at diagnosis.

(A) Patients in age at diagnosis > 50 (initial pCRC vs. second pCRC); (B) Patients in age at diagnosis = 50 - 59 (initial pCRC vs. second pCRC); (C) Patients in age at diagnosis = 60 - 69 (initial pCRC vs. second pCRC); (D) Patients in age at diagnosis = 70 - 79 (initial pCRC vs. second pCRC); (E) Patients in age at diagnosis = 80 - 89 (initial pCRC vs. second pCRC); (F) Patients in age at diagnosis ≥ 90 (initial pCRC vs. second pCRC).

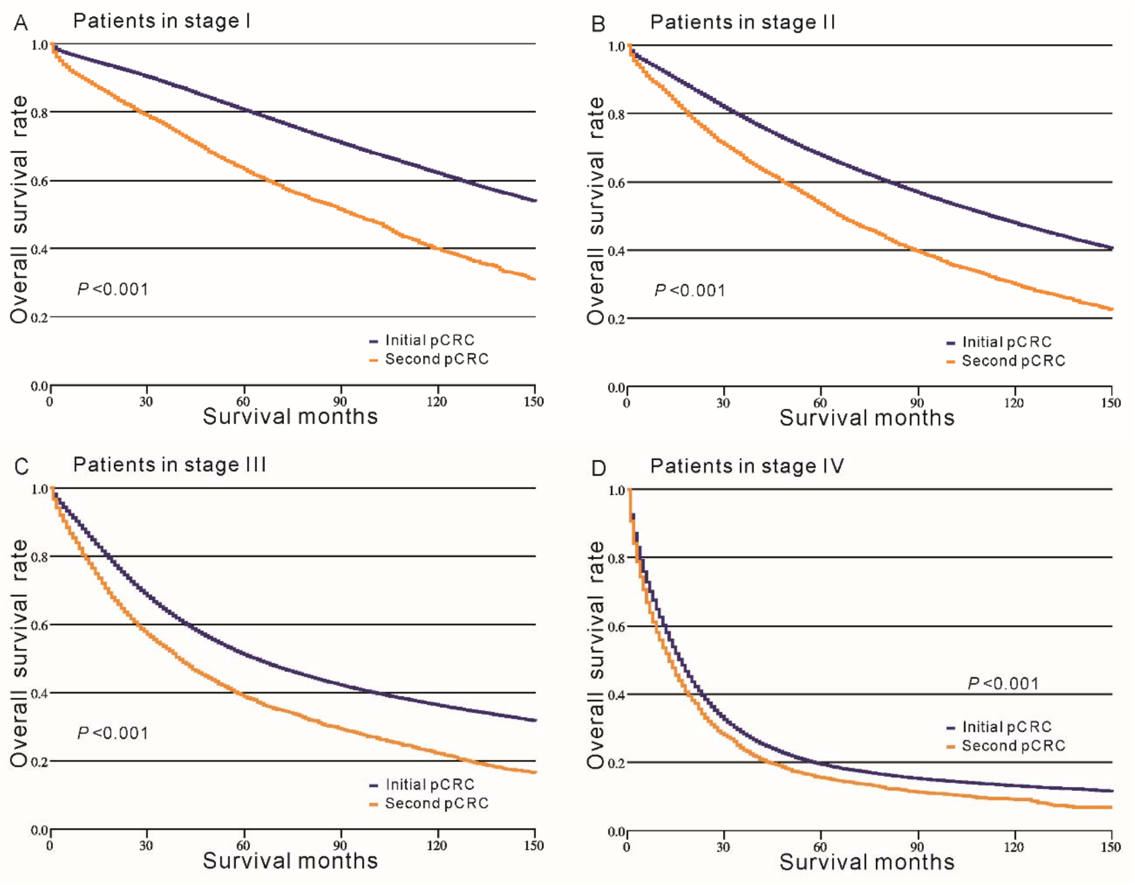

Figure 4 Kaplan-Meier comparison of overall survival among patients with initial pCRC and second pCRC stratified by pTNM stage.

(A) Patients in stage I (initial pCRC vs. second pCRC); (B) Patients in stage II (initial pCRC vs. second pCRC); (C) Patients in stage III (initial pCRC vs. second pCRC); (D) Patients in stage IV (initial pCRC vs. second pCRC).

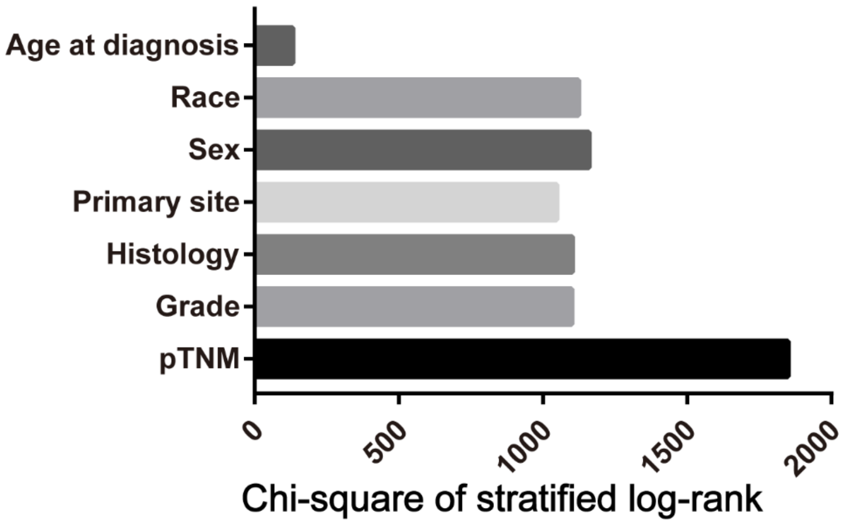

Figure 5 Chi-square of OS log rank after stratified by pTNM stage, grade, histology, primary site, sex, race and age at diagnosis. The Chi-square for Age at diagnosis was lowest.

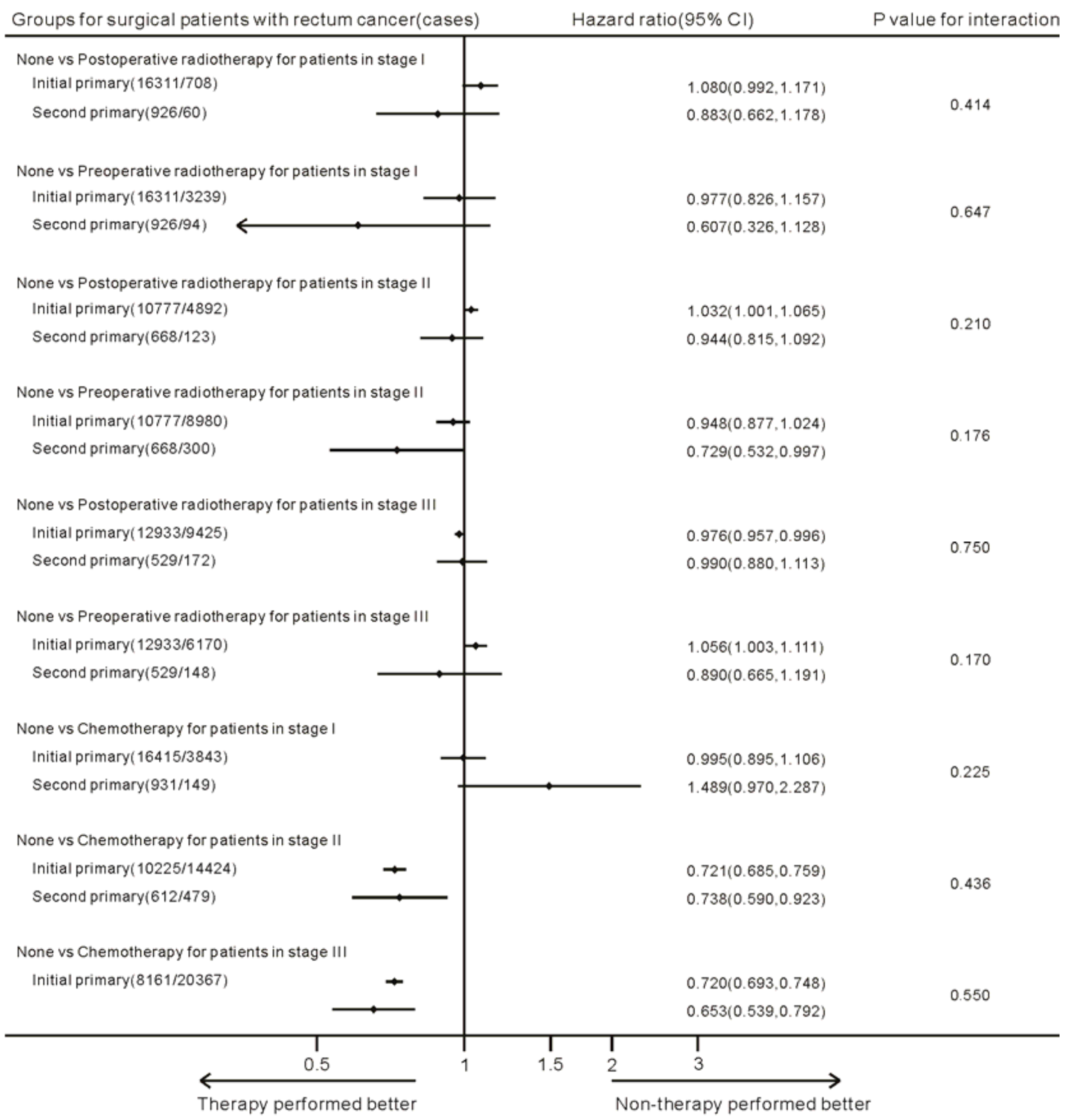

Figure 6 Forest plot of Hazard ratios (HR) and 95% CI in OS different subgroups (Cox proportional hazards model analysis) and interaction comparison between patients with initial and second primary surgical rectum cancer.

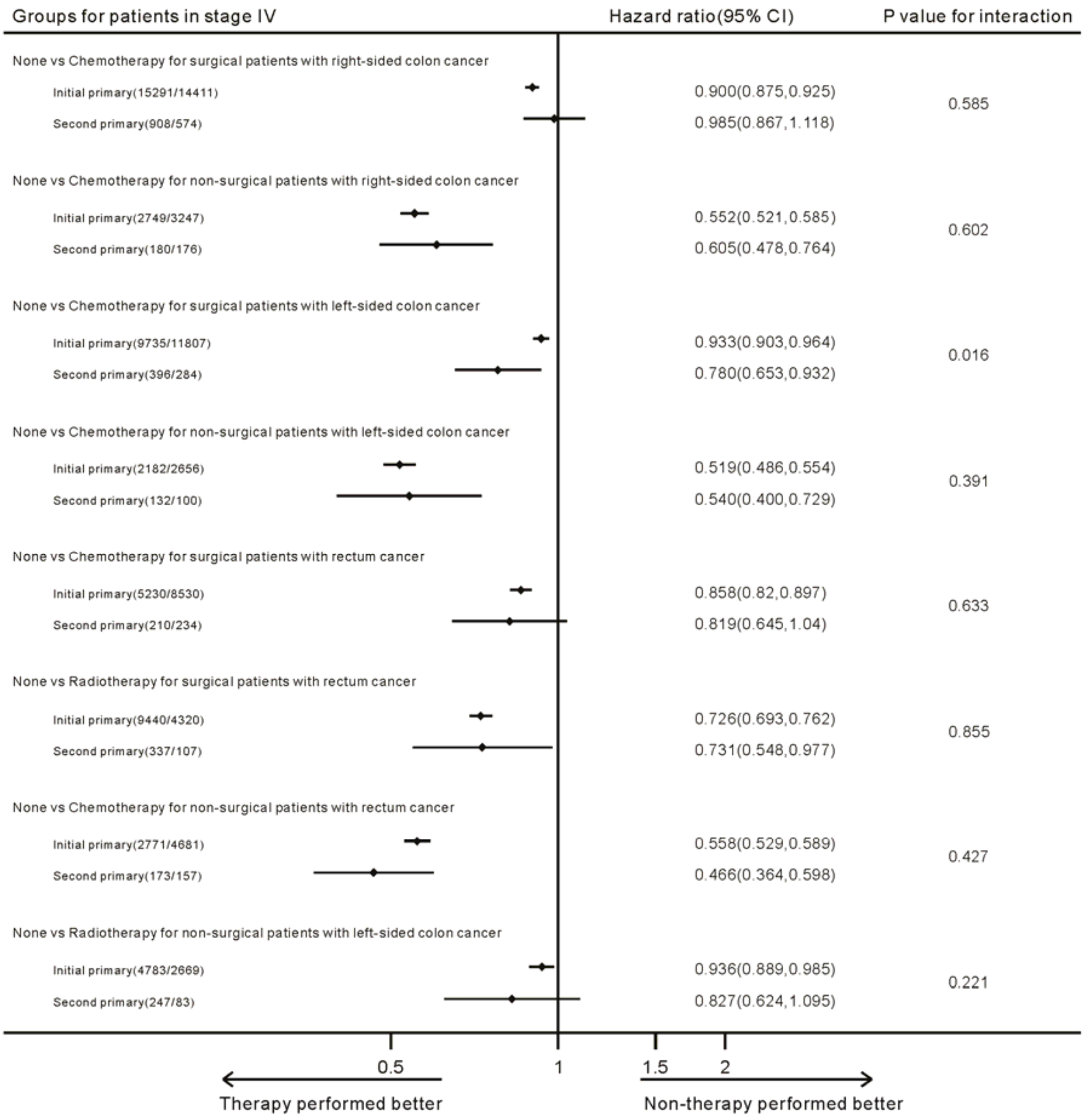

Figure 7 Forest plot of Hazard ratios (HR) and 95% CI in OS different subgroups (Cox proportional hazards model analysis) and interaction comparison between patients in stage IV with initial and second primary CRC.

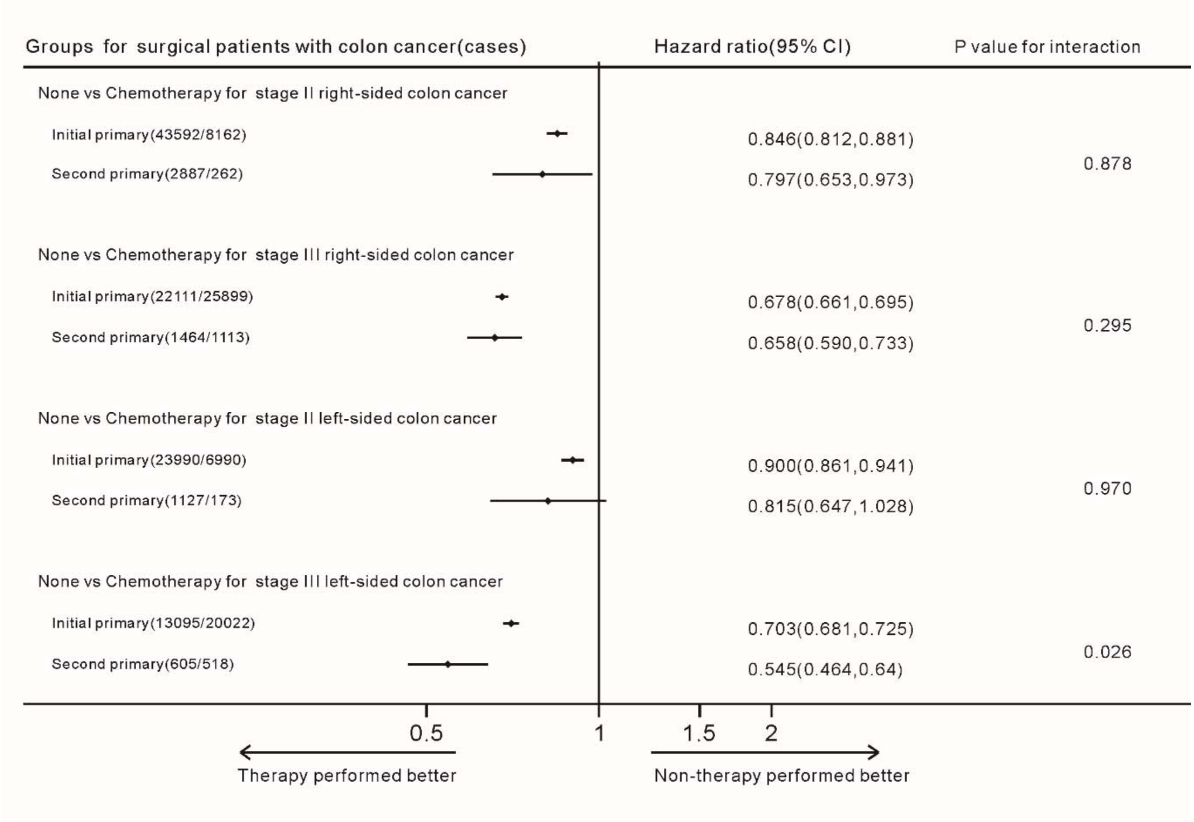

Figure 8 Forest plot of Hazard ratios (HR) and 95% CI in OS different subgroups (Cox proportional hazards model analysis) and interaction comparison between patients with initial and second primary surgical colon cancer.
